# Asymptomatic Huge Cardiac Hydatid Cyst Located in the Interventricular Septum

**DOI:** 10.21470/1678-9741-2018-0368

**Published:** 2020

**Authors:** Taner İyigün, Mugisha Markior Kyaruzi, Veysel Kutay, Seda Tükenmez Karakurt

**Affiliations:** 1 Department of Cardiovascular Surgery, Istanbul Mehmet Akif Ersoy Thoracic and Cardiovascular Surgery Training and Research Hospital, Istanbul, Turkey.; 2 Department of Cardiology, Istanbul Mehmet Akif Ersoy Thoracic and Cardiovascular Surgery Training and Research Hospital, Istanbul, Turkey.

**Keywords:** Cyst, *Echinococcosis*, *Echinococcus*, Cardiopulmonary Bypass, Ventricular Septum, Magnetic Resonance Imaging

## Abstract

The cardiac involvement of hydatid cyst, which is rarely seen, with the location of asymptomatic huge cyst in the interventricular septum (IVS) is an extraordinary condition. We report an isolated cardiac hydatid cyst located in the IVS in an 18-year-old man diagnosed incidentally by transthoracic echocardiography. Cardiac magnetic resonance imaging confirmed a mass lesion of 47×74 mm in diameter located at the base of IVS. The cystic content and its germinative membrane were resected and the cavity was applicated under cardiopulmonary bypass. Postoperative course was uneventful and the patient was discharged on the 6^th ^postoperative day, with oral albendazole therapy.

**Table:** 

Abbreviations, acronyms & symbols	
CT	= Computed tomography
ECG	= Electrocardiogram
IVS	= Interventricular septum
LAD	= Left anterior descending
LV	= Left ventricle
MRI	= Magnetic resonance imaging
RV	= Right ventricle
TTE	= Transthoracic echocardiography

## INTRODUCTION

An isolated cardiac hydatid cyst is very rarely seen. The diagnosis of hydatid cyst can be difficult because of nonspecific variable symptoms depending on the location and the size of the cyst. Cardiac hydatidosis should be suspected in patients with nonspecific symptoms, especially in endemic areas with high occurrence of hydatid infestation. The myocardium of the left ventricle (LV) is more frequently involved, reaching two-three folds more than the right ventricle (RV), with much less involvement of the interventricular septum (IVS)^[1]^.

## CASE REPORT

**Fig. 1:**
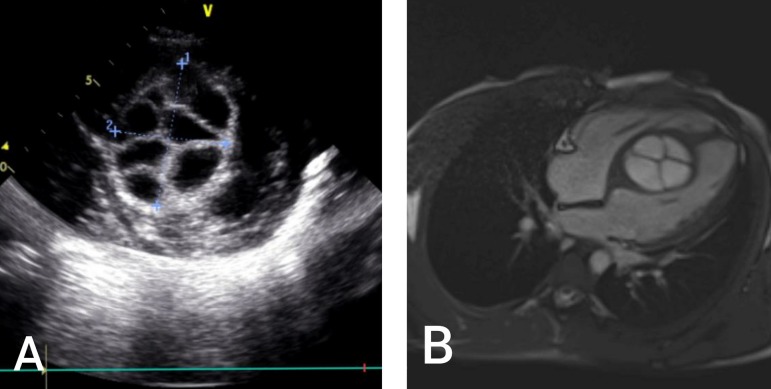
A)Transthoracic echocardiography showing the hydatid cyst in the interventricular septum; B) cardiac magnetic resonance ımaging showing the huge interventricular cyst partially obstructing both ventricles.

An 18-year-old man was referred to a cardiologist for routine cardiac evaluation before attending to the national army recruitment college. He did not show any symptoms of cardiac pathology and did not have history of any health problem. Physical examination was normal. Chest X-ray and electrocardiogram (ECG) were normal. Laboratory analysis revealed negative indirect hemaglutination test for hydatid cyst. Transthoracic echocardiography (TTE) revealed a normal ejection fraction and normal valvular functions with a huge cystic mass within the IVS, leading to partial outflow obstruction of both ventricles (Figure 1A). For detailed identification of the cyst, transesophageal echocardiography was perfomed. A multiloculated huge cystic mass settled at the base of the IVS was detected (Video 1). Cardiac magnetic resonance imaging (MRI) scan confirmed a well-bordered fluid-filled sac containing daughter cysts within the IVS measuring 47×47×74 mm in diameter (Figure 1B). No other cystic lesion was detected in thoracical, abdominal, and cranial scanning by computed tomography (CT).

**Video 1:**
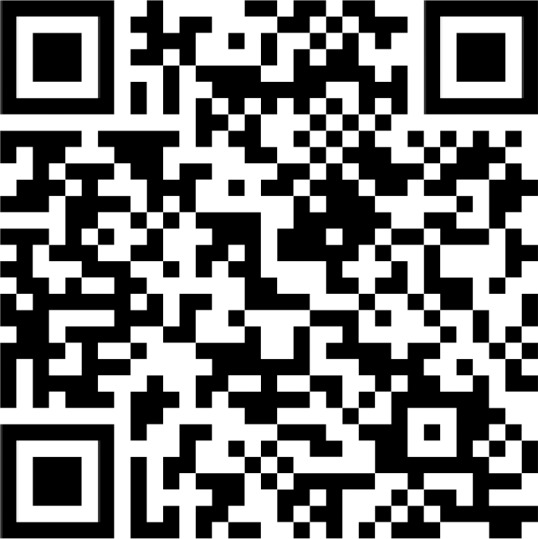
Transesophageal echocardiography showing multiloculated hydatid cyst within the interventricular septum.

**Fig. 2:**
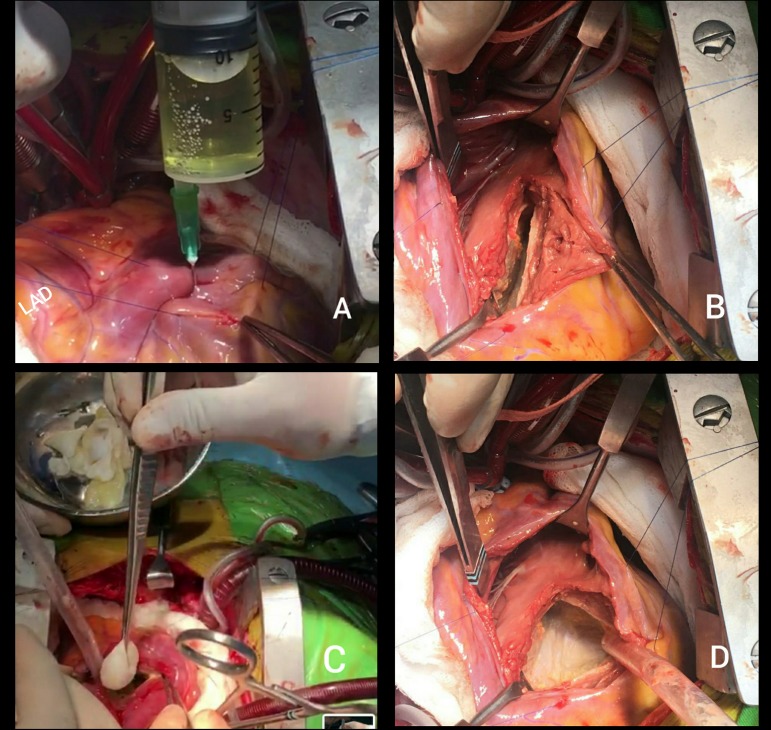
A) Intraoperative photographs showing aspiration of cystic fluid; B) Cystectomy incision; C) Removal of daughter cysts; and D) Completely evacuated cyst cavity. LAD=left anterior descending

The patient underwent surgery under cardiopulmonary bypass. A bulging mass between the RV and LV on the epicardial surface was observed on every heart beat, which indicated the location of the cyst, as diagnosed by MRI and TTE. Pericardium was packed with sterile clean pads soaked in hypertonic saline solution to reduce the risk of mediastinal contamination. In order to reach the hydatid cyst, a vertical right ventriculotomy incision parallel to the left anterior descending coronary artery was made. Fluid of the cyst was aspirated by an injector, then the cavity was irrigated with hypertonic saline solution. Cystectomy was performed, and the germinative membrane and numerous daughter cysts were evacuated (Figure 2). The cystic cavity was plicated (capitonnage procedure) and the myocardial mass of IVS was sutured using Teflon felt strips. After removal of cross clamp, the heart started beating spontaneously in sinus rhythm and was safely weaned from cardiopulmonary bypass. Postoperative course was uneventful, ECG recorded asymptomatic incomplete right bundle branch block. Histopathological analysis of the surgical specimen confirmed the diagnosis of *Echinococcus granulosus*. Postoperative TTE was normal, without any remnant of the cyst. The patient was discharged on the 6^th^ postoperative day, with oral albendazole treatment.

## DISCUSSION

Hydatic cyst is an endemic parasitic disease, mostly occuring in livestock keeping countries. Ingested parasitic larvae migrate through the intestinal mucosa and are mainly carried to the liver by the portal venous circulation^[1]^. Larvae reach the left side of the heart via coronary circulation and the inflammatory response to the presence of parasitic involvement creates an adventitial pericystic layer. Echinococcosis infrequently occupies the myocardium and account for only 0,5%-2% of all hydatid infestations^[1]^. Hydatid cyst of İVS, which has different characteristics from right-sided cysts, is the one with infrequent location of cardiac involvements^[2]^. The presentation of hydatid cyst ranges from asymptomatic to sudden death^[1]^. As it was seen in our case, it may remain silent and is diagnosed incidentally. The clinical picture of the cardiac cyst depends on the location, number of cysts, age, size, and involvement of adjacent structures within the heart. In some cases, left ventricular free wall or pericardial located cysts can be diagnosed easily by simple routine tests, such as an abnormality of the cardiac silhouette on chest X-ray or ECG abnormality imitating left ventricular aneurysmal dilatation^[3]^. Indirect hemagglutination test is specific and a sensitive serologic diagnostic test for hydatid disease with negative result does not exclude the diagnosis.

Unless the pathology is inoperable, all patients who have cardiac hydatid cyst, including asymptomatic patients, should undergo surgery to prevent life-threatening catastrophic complications, such as rupture, anaphylaxia, systemic or pulmonary embolization, arrhythmias, pericardial tamponade, and cardiogenic shock^[4]^. Total excision of the cyst is the best treatment and complete closure of the cyst cavity by plication is mandatory without leading any structural defect. This surgical closure of hydatid cyst has proven to have good results as reported before^[2,4]^.

## CONCLUSION

In conclusion, early diagnosis and urgent surgical intervention are essential even in asymptomatic patients to prevent possible life-threatening catastrophes.

**Table:** 

Author's roles & responsibilities	
TI	Substantial contributions to the conception or design of the work; or the acquisition, analysis, or interpretation of data for the work; final approval of the version to be published
MMK	Substantial contributions to the conception or design of the work; or the acquisition, analysis, or interpretation of data for the work; drafting the work or revising it critically for important intellectual content; final approval of the version to be published
VK	The acquisition, analysis, or interpretation of data for the work; final approval of the version to be published
STK	The acquisition, analysis, or interpretation of data for the work; drafting the work or revising it critically for important intellectual content; final approval of the version to be published
